# Congenital Microcoria: Clinical Features and Molecular Genetics

**DOI:** 10.3390/genes12050624

**Published:** 2021-04-22

**Authors:** Clémentine Angée, Brigitte Nedelec, Elisa Erjavec, Jean-Michel Rozet, Lucas Fares Taie

**Affiliations:** Laboratory of Genetics in Ophthalmology (LGO), INSERM UMR1163, Institute of Genetic Diseases, Imagine and Paris Descartes University, 75015 Paris, France; clementine.angee@institutimagine.org (C.A.); brigitte.nedelec@inserm.fr (B.N.); elisa.erjavec@institutimagine.org (E.E.); jean-michel.rozet@inserm.fr (J.-M.R.)

**Keywords:** congenital microcoria, congenital miosis, dilator muscle, glaucoma, myopia, chromosome 13q32.1 structural variants

## Abstract

Iris integrity is required to regulate both the amount of light reaching the retina and intraocular pressure (IOP), with elevated IOP being a major risk factor for glaucoma. Congenital microcoria (MCOR) is an extremely rare, autosomal dominant disease affecting iris development and hindering both of these functions. It is characterized by absent or underdeveloped dilator muscle fibers and immaturity of the iridocorneal angle—where the aqueous humor is drained—which play a central role in IOP regulation. The dilator muscle anomaly is manifested in pinhole pupils (<2 mm) and thin transilluminable irises, causing both hemeralopia and photoaversion. Axial myopia and juvenile open-angle glaucoma are very frequent (80% and 30% of all cases, respectively). It has been suggested that the immaturity of the chamber angle contributes to glaucoma, and myopia has been ascribed to photoaversion and elevated IOP. Though possible, these mechanisms are insufficient. The disease has been tied to chromosome 13q32.1 structural variations. In addition to compromising iris development, modification of the 13q32.1 architecture could alter signaling pathways for axial ocular length and IOP regulation. Here, we summarize the clinical, histological, and molecular features of this disease, and we discuss the possible etiology of associated anomalies.

## 1. Introduction

**Figure 1:**
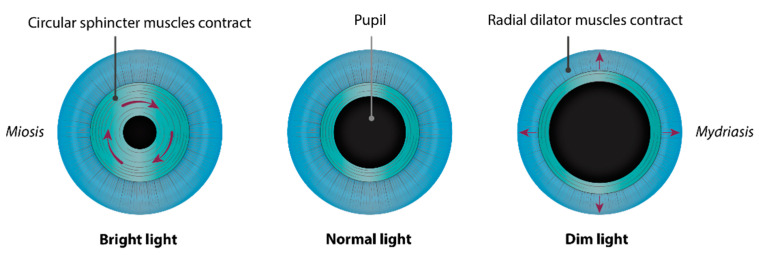
Muscles of the iris and adaptation of pupil aperture to light intensity. The circular sphincter muscle, or pupillary constrictor, near the pupil margin is composed of smooth muscle cells, which can constrict the pupil (*miosis*) when exposed to bright light. It works in opposition to the radial dilator muscle fibers, whose contraction expands the pupillary aperture (*mydriasis*) in a dim environment. The sphincter and dilator muscles are innervated by the parasympathetic and sympathetic nervous systems, respectively.

**Figure 2:**
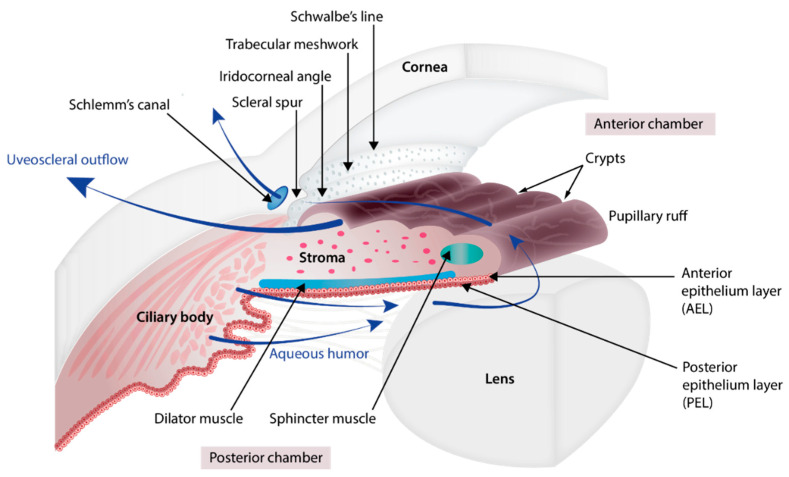
Anatomy of the iris, ciliary body, and aqueous humor pathway. The iris is organized into four layers (from the visible surface layer to the posterior region next to the lens): anterior border layer, stroma, and sphincter muscle, the lightly pigmented anterior epithelium layer (AEL) and dilator muscle, and the heavily pigmented posterior epithelium layer (PEL). The circular sphincter muscle lies within the stroma. The anterior iris epithelium is composed of a single layer of myoepithelial cells. The basal portion of these cells consists in elongated smooth muscle processes forming 3 to 5 layers of radial dilator muscle fibers. The iris root is attached to the ciliary body and to the corneoscleral junction (iridocorneal angle). Near the root, the anterior border layer, stroma, and bilayer epithelium of the iris form finger-shaped processes and become the ciliary body, the anterior and posterior epithelium layers of which are heavily and lightly pigmented, respectively. The aqueous humor that provides nourishment to eye structures is produced by the ciliary body. It flows between the iris and lens, through the pupil, and to the anterior part of the iris, where it runs (i) across the iris and anterior face of the ciliary body, and out through the sclera (uveoscleral pathway) and (ii) through the trabecular meshwork (at the corneoscleral junction with the iris periphery) and out along the inner wall of Schlemm’s canal.

The iris (Figure 1 and Figure 2) is a flat ring-shaped ocular membrane located between the cornea and the lens. Its root is attached to the corneoscleral junction on the anterior side and the ciliary body on the posterior side, next to the lens, and its center is perforated to form the pupil. The iris is composed of a stroma, bilayer epithelium, and two smooth muscles that work in opposition to adapt the pupil aperture to light intensity [1]. The circular sphincter muscle lies within the stroma, near the pupil margin, and can constrict the pupil in miosis [2]. The dilator muscle extends longitudinally within the stroma, from the iris root to below the midpoint of the sphincter [3], and can contract to expand the pupillary aperture in mydriasis [1]. The dilator muscle originates from the anterior iris epithelium, composed of a single layer of myoepithelial cells, the basal portion of which consists in elongated smooth muscle processes [4]. The ciliary body is an extension of the iris, with which it is continuous. It produces a fluid known as aqueous humor that provides nourishment to eye structures. The aqueous humor flows between the iris and lens, through the pupil to the anterior part of the iris, where it is drained through the trabecular meshwork (TM), a sieve-like structure lying at the juncture of the corneoscleral region with the iris periphery [1]. Aqueous humor drainage is required to regulate IOP, which, when elevated, is a major risk factor for optic nerve damage (glaucoma) [5,6].

Congenital microcoria, also known as congenital miosis, is an iris malformation that affects both the regulation of the amount of light reaching the retina and the IOP. It is characterized by partial or total absence of dilator muscle fibers and manifested in pinhole pupils (<2 mm), iris hypopigmentation, and transillumination (Figure 2), causing both hemeralopia and light hypersensitivity [7,8,9,10,11,12]. Juvenile-onset glaucoma, axial myopia, and astigmatism are frequently associated with this condition, which can lead to visual dysfunction or blindness [8]. MCOR is a purely ocular disease. The presence of extraocular symptoms should evoke a differential diagnosis [13,14].

This is a very rare disease. Worldwide, some fifty families have been reported as having the disease, since its first mention about 150 years ago [15]. These cases describe autosomal dominant inheritance, and a unique locus has been mapped and ascribed to 13q32 submicroscopic chromosomal rearrangements [7].

Despite some recent progress, the exact cause of congenital miosis remains elusive, as is the etiology of the associated refraction errors and glaucoma, which is the topic of much discussion. Here, we will review the clinical and histological features of this disease and discuss its possible physiopathology as well as associated anomalies.

## 2. Embryology of the Chamber Angle and Iris

The development of the eye involves the coordinated development of the neuroectoderm, surface ectoderm, and neural crest cell-derived mesenchyme.

The eye begins to develop as a pair of optic vesicles on each side of the forebrain around 3 weeks of gestation [16]. They extend from the forebrain toward the surface ectoderm through the adjacent mesenchyme. The contact of the optic vesicle with the surface ectoderm induces the thickening of the ectoderm, creating the lens placode which invaginates and detaches from the surface ectoderm to form the lens vesicle which will give rise to the lens. Mesenchyme cells begin to migrate into the space between the anterior epithelium of the lens vesicle and the surface ectoderm. The migration continues until the cells condense to form all the layers of the future cornea. The primitive corneal endothelial layer and future trabecular meshwork are formed from posterior mesenchyme cells whereas the surface ectoderm that covers the anterior side of the mesenchyme will become the corneal epithelium. In between, the mesenchyme cells differentiate to form the corneal stroma. During differentiation of the corneal endothelium, the lens detaches from the future cornea, creating the anterior chamber cavity [17].

Whilst the lens vesicle is forming, the optic vesicle also invaginates to form the double-walled optic cup. This iris and ciliary body derive from both the neuroectoderm and mesenchyme [18]. The epithelial layers of the iris and ciliary body, like the retina, develop from the third month of gestation as an outgrowth of the optic cup whilst the lens and the cornea are being formed. The outer wall produces pigment and forms the retinal pigment epithelium (RPE) and the inner wall differentiates to form the neural retina. The developing RPE and retina meet at the anterior rim of the optic cup, close to the lens vesicle which induces the differentiation of the cells of the inner wall of the anterior optic cup into the posterior pigmented epithelium of the iris (continuous with the developing retina) and the cells of the outer wall form the anterior iris epithelium (continuous with the developing RPE) [19]. At the root of the iris, the epithelium layers fold and the cells differentiate further to form the ciliary process epithelium [1]. The stroma of the iris and ciliary body arise from mesenchymal cells that migrate to the angle between the future cornea and the anterior edge of the optic cup which begins to extend to form the iris. The cells proliferate and migrate along the iris and ciliary body epithelial layers in formation and differentiate into stromal cells. Within the stroma of the iris, the sphincter pupillae and dilator pupillae muscles develop from optic cup neuroectodermal cells, contrasting with the ciliary muscle which derives from the mesenchyme [2]. The sphincter muscle arises from outer wall of the rim of the optic cup [20]. A group of cells characterized by diminished melanogenesis are distinguishable and indicate the future sphincter which begins to develop at 4 months and is well formed by 6 months [21]. The dilator pupillae also develops from the outer wall of the optic cup, but in a slightly more peripheral location than the sphincter. At the 6th month, myofilaments of the dilator muscles began to appear in the cytoplasm of the peripheral anterior pigment epithelium layer, with villous protrusions toward the stroma. The dilator muscle is fully formed histologically in human fetuses at eight months [22].

In parallel to the formation of the iris, the mesenchyme cells that migrated at the chamber angle separate from each other, generating small open spaces filled with extracellular fibers which will further organize into trabecular beams and vessels form close to the sclera which will ultimately form Schlemm’s canal [17]. Just posterior to the canal, tissue condenses to form the scleral spur that is composed of collagen and is continuous with that of the trabecular beams [5,23]. Between the trabecular beams and Schlemm’s canal, some mesenchymal cells differentiate into endothelial cells and fibroblasts which are embedded in a matrix of collagen, elastic-like fibers, and ground substance, forming the juxtacanalicular tissue [24]. While the TM is forming, the anterior chamber expands and its peripheral margin slides posteriorly, exposing the TM to the chamber cavity. The structures involved in aqueous humor drainage develop late, being mature around birth, and the anterior chamber is defined at 5 months of gestation [17].

## 3. Disease Description

The first mention of the disease dates back to 1862, when W. R. Wilde reported a series of three unrelated individuals displaying pinhole pupils without neurological problems, a condition he called “miosis congenita” [15]. In the following years, a dozen similar observations were reported by a few other ophthalmologists [25,26], some of whom described weakened pupil response to mydriatics [27]. To the best of our knowledge, a total of 160 affected individuals from 49 families have to date been reported with a bilateral disease characterized by partial or total absence of pupil dilation, even after mydriatic treatment [8,9]. A featureless surface with poorly developed collarette and crypts, reduced iris pigmentation, iris stroma thinning, and visible transillumination are typical of the disease [9,28,29,30,31,32].

Defective development of the iris musculature was suspected early on, as indicated by the phrases “fault of development” and “feeble development of the iris musculature” found in initial descriptions of the disease [16,17]. This was substantiated in 1923 by a Norwegian ophthalmologist and pathologist, who provided a detailed clinical description of the ocular phenotype in three siblings combined with a post mortem anatomical analysis of the eyes of two of them who had died of apoplexia cerebri [33]. This study, along with subsequent post mortem analyses of irises and iridectomy specimens from 25- to 72-year-old individuals, revealed significant iris thinning with atrophy of the stroma, displaying a normal ultrastructure and abundant collagen fibrils but greater numbers of fibroblasts and melanocytes in the ground substance [11,28,32,34,35,36,37]. Consistent with the observation of partial or total dilation inability among affected individuals, varying dilator muscle anomalies have also been described—from peripheral to generalized dearth or absence of stromal contractile processes of the anterior iris epithelium [8,9,10,11,21,34,35,38,39]. Existing myofibrils can be normal in appearance, particularly behind the sphincter muscle and in the intermediary region [33], or greatly disordered and lack myofilaments and desmin [9,28,33,34,35,37]. Thickened fibrobrotic and vacuolated dilator muscle can be observed [37]. The sphincter muscle and the ciliary body are normal, as are the innervation and vasculature [32,34,40]. In humans, the iris and ciliary body epithelia develop from the third month of gestation as an outgrowth of the anterior margins of the neuroectoderm-derived optic cup [41,42]. At 16 weeks of gestation, there is a distinct pigmented bilayer epithelium at the site that will later accommodate the adult iris. Myofilaments appear in the posterior iris epithelium near the presumptive pupillary margin in the 10th week, and in the cytoplasm of the peripheral anterior pigment epithelium in the 6th month, forming histologically recognizable sphincter and dilator muscles in the 6th and 8th months, respectively [43,44]. Differentiation of the anterior layer of the iris epithelium is manifested by the expression of α smooth muscle actin in the 28th week, and desmin intermediate filaments in dilator fibers by the 37th week [45]. Histological observations of all dilator muscle developmental stages, sometimes for the same iris [33]—ranging from a normal structure behind the sphincter, through poor differentiation with sparse and highly disordered fibers lacking myofilaments and intermediate filaments [9,10,37,46], down to complete growth inhibition [11,34]—suggest anomalies in the terminal stages of anterior iris pigment epithelium differentiation. Furthermore, this observation, which correlates with the variability of dilation phenotypes (partial or absent dilation ability), suggests that the genetic defect underlying the disease has a stochastic effect on development of the eye.

### 3.1. Associated Signs

#### 3.1.1. Glaucoma

Cases of glaucoma in individuals with congenital miosis were sporadically reported in 1949 and the following decades [40,46,47,48]. Studying over 40 members of a Breton family spanning five generations—the oldest of whom were evaluated in the 1960s [49], Toulemont and coll. Toulemont, P.J. et al showed that glaucoma was significantly associated with microcoria, having been observed in seven out of 23 microcoric individuals, but none of the unaffected relatives (Fisher’s exact test: *p* = 0.001) [8]. Similar observations were made for a four-generation Indian pedigree comprising 18 individuals with congenital miosis, 11 of whom had glaucoma [30] and a three-generation Japanese family of which four out of five members had glaucoma [9]. In total, glaucoma or high IOP has been reported in association with congenital miosis in 36 individuals from eight families [8,9,10,30,31,40,46,47,48,50,51,52], whereas absence of glaucoma is mentioned in 71 patients. Diagnoses were made as early as ages seven [8,10] and 12 [30], but mostly in individuals in their early twenties [8,9,10,30,31,50] or thirties [9,30,47,51] (median age at diagnosis = 25 years). Glaucoma has been reported in some older persons, including two relatives aged 40 and 55 belonging to the second generation of the four-generation Indian pedigree. In this family, nine other individuals with congenital miosis had glaucoma in their second or third decade, suggesting late diagnosis in earlier generations. Late-onset glaucoma has been described in a 64-year-old from a three-generation family including eight individuals with congenital miosis [46]. This individual may have had chronic simple glaucoma, as seen in another case involving a 59-year-old [40].

Typically, glaucoma in this condition is characterized by elevated IOP, up to 60 [8] and 70 mm Hg [9] (mean: 29 mm Hg, SEM: 2.43 mm Hg versus 16.3 mm Hg, SEM: 0.62 in counterparts with no glaucoma [8,9,30,40,50]; unilateral *t*-test: *p* < 0.00001). Several authors have emphasized the difficulty of monitoring glaucomatous damage to the optic nerve due to pupillary miosis, which complicates fundus examination, and high myopia, which modifies the shape of the optic disk [8,28].

The etiology of glaucoma in congenital miosis is obscure. Occasionally, gonioscopic examination has revealed shallow anterior chambers and narrow angles, which might contribute to glaucoma [40,47]. The vast majority of individuals with both congenital miosis and glaucoma display wide-open chamber angles, although with prominent iris processes expanding over the TM and, in most cases, a high insertion of the iris root into Schwalbe’s line, the TM, or the scleral spur [8,9,46,50,51]. Prominent iris processes are not uncommon in the general population and are not believed to have any influence on IOP regulation [30,31]. However, some authors consider that the additional chamber anomalies might compromise aqueous humor outflow, and they describe open-angle goniodysgenetic [46] or developmental glaucoma [9]. These designations have been widely debated because—as mentioned by Toulemont and colleagues [8]—the chamber angle anomaly associated with congenital miosis cannot be categorized into a known iris dysgenesis subclass of the Anatomical Classification of the Developmental Glaucomas by Hoskins et al. [53]. More importantly, it should be noted that the same gonioscopic anomalies are very common in microcoric individuals with normal IOP, even at advanced ages (seven out of eight [46]; 16 out of 27 [8]). In the absence of precise information on etiology, the term *childhood-onset*, or *juvenile glaucoma* is preferable [8,51].

A normal chamber angle is rather uncommon in individuals with congenital miosis. However, it is interesting to note that the review of these cases highlights the consistent absence of glaucoma [11,21,32,34,38,54]. This observation suggests that angle anomalies are necessary but not sufficient to trigger IOP elevation and glaucoma. The extracellular matrix (ECM) of the TM is thought to have an important role in the regulation of IOP [55]. Interestingly, histopathologic examination of TM biopsies from two brothers with elevated IOP belonging to the three-generation Japanese MCOR family showed thickened and fibrotic connective tissue in the juxtacanalicular region, with accumulations of ECM [9]. This observation suggests a role of ECM homeostasis in congenital miosis-associated glaucoma.

#### 3.1.2. Axial Myopia

Myopia as another contributing factor for developing or aggravating glaucoma in congenital microcoria is possible. Population-based studies indicate that myopia is strongly associated with glaucoma in both adults [56] and children [57] and that the risk of glaucoma increases with an increasing degree of myopia [56,58,59]. Furthermore, some studies have demonstrated that elevated IOP during postnatal eye growth can increase the length of the eye [8,60].

Axial myopia is extremely frequent in congenital miosis. It was mentioned in the initial description of the disease by R. Wilde, who noted that one of those studied was “remarkably near-sighted” [15]. In 1986, Mazzeo and colleagues reported several cases of individuals with congenital miosis and myopia, but they were born from a myopic mother, which casts doubt on an association between the traits [46]. Like glaucoma, myopia was demonstrated to be correlated with congenital miosis in the multigenerational Breton family (Fisher’s exact test: *p* < 0.0001) and was also prevalent in the Indian (15 out of 18 microcoric individuals were myopic [30]) and Japanese (five out of five considered [9]) families. The review of all the cases indicates that at least 70% of individuals displaying congenital microcoria were myopic, half of whom suffered from glaucoma, knowing that 95% of individuals suffering glaucoma were myopic [21,30,33,35,39,49]. Of note, in the three-generation Indian family [29,30], glaucomatous subjects displayed higher refraction errors (mean: −12.5 Diopters (D); SEM: 4.67 D) than their counterparts with normal IOP (mean: −2 D; SEM: 0.41 D; Wilcoxon test: *p* = 0.040). Similarly, Toulemont and colleagues noted that, in the five-generation Breton family, microcoric individuals with glaucoma had high myopia, ranging from −4 to −19 diopters [8]. Elevated IOP in some of these cases may play a role in axial elongation of the eye. However, myopia is also frequent in individuals with congenital miosis and normal ocular tension (57% of the cases for whom both IOP and refraction information were available) [21,30,33,35,39,49]. For example, an individual from the multiplex family that was described by Holth and Berner in 1923 presented with high myopia at age 30 (−7 D, −8 D) and an even higher refraction error at age 52 (−15 D, −16 D) but normal IOP, leaving the mechanisms behind axial length elongation in congenital miosis an open question [33].

Some authors have proposed that propensity to close the eyelids to reduce glare caused by iris transillumination in congenital miosis could elicit a form-deprivation myopia. Indeed, monoocular myopia in infants with unilateral eyelid closure [61,62,63] and axial myopia following surgical eyelid closure at various points of postnatal development in primates and mice have demonstrated that ocular occlusion can increase the axial length of the eye [64,65,66]. However, the lack of correlation between myopia and oculocutaneous albinism, where iris transillumination and photoaversion are major symptoms, challenges these assumptions.

#### 3.1.3. Astigmatism and Other Corneal Anomalies

Corneal anomalies have been reported by several authors, the most frequent being astigmatism, present in 51 subjects with congenital miosis, among the 63 whose data are available from the literature [8,9,12,21,28,33,35,36,39,40,46,54,67]. Of these individuals, data available for 16 of them indicated the pattern of astigmatism as being with-the-rule (WTR) astigmatism in the majority of eyes (73%) and against-the-rule (ATR) astigmatism in 19% of eyes [12,28,33,35,36,39,40,46]. The association is statistically significant in the five-generation Brittany family: an anterior corneal astigmatism of >0.5 D was observed in 20 out of 23 microcoric individuals—1.75 to 4.00 D for 12 of these 20 (52%)—compared with only 20% of control subjects (Fisher’s exact test: *p* < 0.0001) [8].

Other corneal anomalies described include corneal edema [9,40] and megalocornea [21,39]. Megalocornea has reportedly affected all microcoric subjects from two-generation (two subjects) and three-generation (six subjects) pedigrees [21], a two-generation family (two subjects) [39], and a sporadic case [68]. Although rather infrequent (11 individuals in four families), the association of the two ocular anomalies may not be random. It would be interesting to determine whether the molecular cause of the disease in these subjects differed from that for affected individuals in other families.

#### 3.1.4. Cataract

There have been 12 cases of late-onset or senile cataracts [11,21,28,67] and two cases of congenital cataracts [25,34] reported in individuals with congenital miosis. Their occurrence is likely coincidental considering that neither senile nor congenital cataracts are more prevalent in microcoric subjects than in the general population.

## 4. Genetics

At least 24 multigenerational pedigrees (>100 cases in total) have been reported, while there have only been a dozen sporadic cases. Dominant transmission was first proposed in 1949 [69] and further supported by the initial description of the Breton pedigree [49]. Retrospective analysis of published pedigrees reveals a moderate predominance of affected males (84 men, 63 females; M/F ratio: 1.3), but there is no difference in clinical presentation between the sexes. The disease is transmitted equally by males and females, and father-to-son transmission is not uncommon (at least 25 occurrences [10,29,61]), demonstrating autosomal inheritance. In multigenerational pedigrees, segregation analysis indicates the absence of disease transmission through unaffected obligate carriers, suggesting complete penetrance of the disease [8,9,10,29,30]. It should be noted that, in 1979, Polomeno and Milot suggested autosomal recessive transmission in two sporadic cases concerning individuals born to consanguineous parents [21]. However, today it is well known that dominant de novo mutations are not uncommon in consanguineous families [70,71].

In the late 1990s, Rouillac and colleagues applied whole genome-linkage analysis to the five-generation Breton family, mapping the disease locus to chromosome 13q31-32, in an 8 cM interval flanked by markers D13S1239 proximally and D13S1280 distally [10]. Analysis of the large four-generation Indian pedigree matched the region of interest to a 4 Mb interval between markers D13S265 and D13S1280 [30]. The genetic heterogeneity of the disease has been hypothesized on the basis of primary mapping analysis in two unrelated British families, one of which reportedly did not exhibit a defect at this locus [38]. However, as highlighted by Ramprasad and colleagues in 2005, the markers at or flanking the locus were not analyzed, and therefore linkage cannot be ruled out [30].

**Figure 3:**
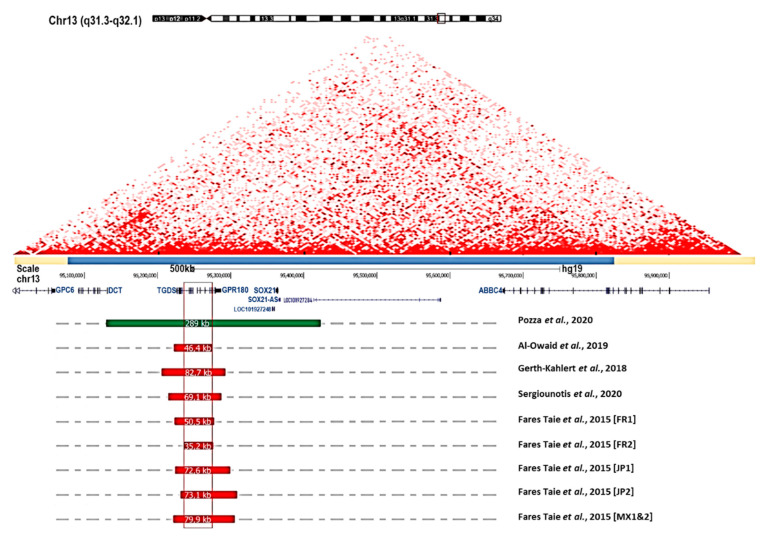
Summary of 13q32.1 structural variations associated with congenital microcoria. The two-dimensional heat map representing normalized Hi-C sequencing visualization in HMEC with the 3D Genome Browser is shown [72]. The topologically associating domain (TAD) encompassing the MCOR region and spanning 600 kb is indicated with a light blue bar (chr13:95,075,000–95,825,000, Hg19 and 5 kb resolution). Gene organization at chromosome 13q32.1, and position and size of structural variations (deletions in red; duplication in green) reported in individuals suffering from congenital microcoria [7,31,32,67]. The critical deletion (shown in red frame) responsible for MCOR spans 31.8 kb (hg19, chr13: 95,241,606–95,273,292). The reciprocal duplication encompasses 7 genes: *DCT, TGDS, GPR180, LOC101927248, SOX21, SOX21-AS*, and *LOC101927284* [12].

The very high LOD score obtained by primary mapping for the five-generation Breton family (*Z*_max_ = 9.79) unambiguously mapped the disease to 13q31-32 [10]. Yet, Sanger-based mutational screening of genes lying within the genetic interval failed to detect the disease-causing mutation [7]. Thus, array comparative genomic hybridization was used to search for structural variations, revealing a submicroscopic 54.8 kb heterozygous deletion in the family, as well as 79.9 kb, 72.6 kb, 35.2 kb, 79.9 kb, and 73.1 kb overlapping deletions in the three-generation Mexican [28] and Japanese [9] pedigrees and three previously unreported families of French, Mexican, and Japanese origin, respectively [7]. Subsequently, four additional structural variations involving the same region were published. Deletions of lengths 69, 82, and 46 kb were reported in multigenerational families from the UK [31], Switzerland [32], and Saudi Arabia [67], respectively. In the British family, which comprised five affected subjects over three generations, iridocorneal angle anomalies were reported to affect at least three individuals aged >40, of whom two (mother and son) had juvenile glaucoma [31]. In the other two families, disease description was limited to a mother and two children. All were described as having normal chamber angle and early onset myopia [32] or minimal hyperopic astigmatism [67]. The mother in the Saudi family reportedly suffered bilateral senile cataract [67]. All the deletions except the one identified in Mexican families differ from each other, which suggests independent events [7]. Extensive bioinformatic analysis of deletion breakpoints and surrounding genomic architecture has identified a duplicated sequence prone to recurrent nonallelic homologous recombination in one case. The others likely involve nonrecurrent microhomology-mediated rearrangement [7]. Analysis of the positions of the deletions, which range in size from 35 kb to 85 kb, places the critical congenital miosis-causing deletion in a 31.8 kb interval (Figure 3). Although variable in size, the deletions encompassed or interrupted the tail-to-tail genes *TGDS* and *GPR180*.

Recessive mutations in *TGDS*—encoding TDP-glucose 4,6-dehydratase—cause malformations of the mouth, face, and digits (Catel–Manzke syndrome (CATMANS); MIM: 616145). Ophthalmologic examination of individuals with *TGDS*-associated CATMANS at Necker Hospital (Paris, France) revealed no iris anomalies, suggesting *TGDS* does not play a role in MCOR. *GPR180*—encoding G protein-coupled receptor 180—is involved in the regulation of smooth muscle cell growth. However, analysis of knockout mice and individuals from a two-generation family carrying a heterozygous *GPR180* nonsense mutation failed to detect any iris dilator muscle anomaly. Yet, those family members (aged 16 to 62) harboring the *GRP180* loss-of-function mutation displayed prominent iris processes in the chamber angle expanding over the TM, and normal IOPs [7]. This observation suggests that *GRP180* loss-of-function may compromise iris development, but is not sufficient to cause congenital miosis. The disease likely involves the loss of elements regulating the expression of genes neighboring the deletions. The deleted sequences are contained in a topologically associated domain (TAD) spanning 600 kb on chromosome 13q32.1. The deletions may change the enhancer dosage, resulting in the loss of function of one or more of the 8 genes in the TAD or result in upregulation or tissue-specific misexpression [12]. *DCT* encoding daupachrome tautomerase is among the genes of interest. It is involved in the biosynthesis of eumelanin [73]. Recently, biallelic loss-of-function mutations in *DCT* have been described in oculocutaneous albinism (OCA8) [74]. Misexpression of this gene may contribute to the disease or at least the excessively light pigmentation of the iris reported in subjects with congenital miosis. *SOX21* encodes a transcription factor of the SRY-related HMG-box (SOX) family which is transiently expressed during the early phases of optic vesicle morphogenesis in chicks and during specification in the lens and retina but is switched of afterwards, i.e., before the iris starts developing [75]. Its loss of function in chicks, as in zebrafish, interferes with normal lens development [76]. Ectopic expression during iris development could be induced by 13q32.1 deletions and therefore compromise the development of the iris and chamber angle. Finally, *ABCC4* (*MRP4*) immunoreactivity has been demonstrated throughout the aqueous outflow pathway, including the trabeculum meshwork, Schlemm’s canal, and juxtacanalicular tissue [77]. This gene has been suggested to regulate intracellular cAMP and cGMP levels in the TM and IOP, possibly through the regulation of ECM homeostasis. Whether misexpression of this gene contributes to congenital miosis-associated glaucoma merits consideration. However, in the five-generation Breton family, 29 of the 31 MCOR patients shared a 4 Mb haplotype encompassing the 600 kb TAD (markers D13S1300 and D13S154) at the 13q32 locus [10]. As myopia and glaucoma were not consistent features among the 29 haploidentical individuals, and as crossovers are highly unlikely in a 4 Mb interval, it is unlikely that structural variants are directly involved.

Interestingly, a recent study has reported a reciprocal 289 kb duplication encompassing seven genes (including *TGDS* and *GPR180*)—identified in a mosaic mother and her daughter with congenital miosis and a normal anterior chamber angle [12]. From this observation, the authors attributed the chamber angle dysgenesis identified in families carrying 13q32.1 deletions to partially non-functional GPR180. However, this is challenged by the report of normal chamber angles in the Swiss and Saudi Arabian families carrying deletions encompassing *GPR180* [32,67] (Figure 3). The duplication is contained in the 600 kb TAD and may result in dose-dependent upregulation or tissue-specific misexpression [12].

## 5. Discussion

Congenital microcoria was initially described over 150 years ago and, as cases were reported, additional ocular anomalies were noted. While the rarest of these may be coincidentally present, others like myopia, astigmatism, and glaucoma are correlated. Thus far, the identification of structural variations as the cause of the disease has not made it possible to unravel the mechanisms underlying the observed iris developmental defects. The association with refraction errors or glaucoma appears to be independent of the size or type of structural variation, and whether they are secondary to the iris anomalies or the modification of the regulatory architecture of chromosome 13q32.1 remains a mystery. This review suggests that the combination of iris and chamber angle dysgenesis and genetic anomalies is necessary for glaucoma to occur in congenital microcoria. Photoaversion and immaturity of the chamber angle could contribute to myopia and elevated IOP, which may itself aggravate myopia. However, the absence of an increased prevalence of deprivation myopia in oculocutaneous albinism and the higher prevalence of myopia among individuals with normal IOP make the causative mechanism uncertain. This review highlights that chamber angle anomaly is common both in glaucomatous and nonglaucomatous individuals. Yet, while all glaucomatous individuals display angle anomalies, none of the few people with normally developed chamber angles had glaucoma, suggesting that the angle anomaly is necessary but insufficient to trigger glaucoma. The report of ECM accumulation in the TM of two siblings may provide a clue. There is increasing evidence that ECM accumulation in the juxtacanalicular region, the deepest part of the TM, is a main trigger of open-angle glaucoma, including primary open-angle glaucoma. It reduces the filtering capacity of the TM and increases aqueous humor outflow resistance, leading to elevated IOP and, ultimately, the death of retinal ganglion cells, whose axons form the optic nerve [24,78]. It is probable that high IOP in congenital miosis is due to the combination of chamber angle anomalies and deregulated ECM homeostasis. In addition to compromising the proper development of the iris, it is possible for 13q32.1 structural variations to deregulate signaling pathways important for axial length control and for ECM turnover in the TM.

## 6. Conclusions

Despite the identification of chromosomal rearrangements in individuals suffering from congenital miosis, the mechanisms underlying the defective development of the iris and chamber angle and the cause of associated glaucoma and refraction errors remain elusive. Further studies, including whole-genome sequencing with Hi-C sequencing in multigenerational families in the search for genetic factors increasing susceptibility to glaucoma and myopia, will hopefully shed light on the intriguing mechanisms behind these ocular anomalies and how they are linked to each other.

## Data Availability

Not applicable.
